# Editorial: Debates in elite sports and performance enhancement: 2022

**DOI:** 10.3389/fspor.2023.1223531

**Published:** 2023-06-06

**Authors:** Marco Beato, Joseph S. Marino, Antonio Dello Iacono

**Affiliations:** ^1^ School of Health and Sports Sciences, University of Suffolk, Ipswich, United Kingdom; ^2^ Department of Applied Physiology, Health, and Clinical Sciences, University of North Carolina, Charlotte, NC, United States; ^3^ School of Health and Life Sciences, University of the West of Scotland, Glasgow, United Kingdom

**Keywords:** soccer, science, research, debate, sport, performance

**Editorial on the Research Topic** Debates in elite sports and performance enhancement: 2022

## 1. Introduction

Success in sport depends on research and the advancement of applied science to ensure health and achieve performance enhancement among athletes. However, to further progress evidence-based practices, there is a need to critically revise and discuss around emerging challenges that sport practitioners face. Frontiers in Sport and Active Living recognizes the importance of facilitating debate and discussion amongst the community, and so has organized this Research Topic, offering a platform for such discussion to occur in the field of sports science. This Research Topic entitled “Debates in Elite Sports and Performance Enhancement” aimed to highlight themes that foster debate and discussion in the high-performance sports industry. We encouraged authors to submit opinions or perspective pieces that focus on the “pros” or “cons” of all areas surrounding the athlete's environment for performance enhancement. Our final goal was to respectfully challenge perspectives and paradigms to enhance sports performance.

## 2. Articles

This Research Topic of Frontiers in Sports and Active Living, “Debates in Elite Sports and Performance Enhancement, 2022” contains 10 original manuscripts meeting the editorial criteria, including one original research article (McCaskie et al.), three opinion piece articles (Beato, Desroches and Goulet, Lerebourg and Coquart), one review (Flack et al.), one systematic review (Gualtieri et al.), two brief research reports (Beato et al., Beato et al.), and two perspective articles (Baker et al. Johnston et al.).

McCaskie et al. studied the relationship between pre-season body composition, in-season match performance, and match availability in female players competing in the Australian Football League Women's competition. This study found that body composition characteristics are not able to differentiate higher versus lower performing players.

In an opinion article, Beato provided recommendations in the field of research design, specifically, randomized controlled trials and data interpretation. The author aimed at improving the robustness of future strength and conditioning research (e.g., training, performance, injury prevention) and avoiding the replication of common mistakes such as the use of inadequate sample sizes, type 1 and type 2 errors, incorrect interpretation of confidence intervals, the use of magnitude-based inference, and the use of within-group comparisons instead of between-group comparisons to determine longitudinal differences between intervention.

In their opinion article, Desroches and Goulet elaborated on the challenges and future directions of the Ironman™ triathlon, an ultra-endurance event consisting in 3.8 km swimming, 180 km cycling and 42.2 km running. In particular, by the means of predictive analyses, they elucidated whether an Ironman™ distance triathlon under 7-h might be achievable in 2022 without external assistance and with the current swimming, cycling, and running equipment. Moreover, they discussed how external aid should be deployed to achieve the breaking of the 7-h mark.

In their opinion article, Lerebourg and Coquart critically analyzed the “ideal” running pattern to optimize running performance, while taking into consideration the multifactorial aspects of performance (i.e., physiological, biomechanical, psychological, environmental, and technological factors). In view of the multifactorial nature of performance, they encouraged turning to Big Data and Artificial Intelligence approaches to facilitate the programming components (i.e., planning, implementing, monitoring, predicting) of sports performance.

Flack et al. reviewed articles describing the possible interaction between bilirubin and exercise and the expected effects on metabolic health and athletic performance by describing the underlying mechanisms at hormonal and cardiovascular level.

The systematic review of Gualtieri et al. provided a comprehensive summary of the evidence about absolute and individual velocity thresholds used to classify high-speed running and sprinting demands in soccer, competitive standards, and lastly, strategies for eliciting high-speed running and sprinting during training in professional adult soccer players.

In their brief research report, Beato et al. compared the external and internal training load demands of sided-game drills in professional soccer players during the official season. While sided-games can replicate or even exceed some match-specific intensity parameters, high-speed running and sprinting distances were consistently lower compared to official matches. These findings can inform practical applications for soccer practitioners.

In another brief research report, Beato et al. compared internal and external loads between different between sided game formats, and assessed whether positional differences exist in professional soccer players. Overall, different formats may be used to induce selective responses (e.g., accelerations, rate of perceived exertion), with playing position influencing external load metrics, such as high-speed running and decelerations but not rate of perceived exertion and distance.

Baker et al. in their perspective article, highlighted several discrepancies in the way development is conceptualized, contextualized, and operationalized between different competitive levels (e.g., pre-professional sport and professional sport). They used available evidence to provide guidance for researchers and practitioners to encourage the delivery of structured developmental programming in professional sport systems to aid with the transitionary period between pre-elite and elite levels, and to help foster career longevity.

In their perspective article, Johnston et al. warranted the need for researchers and practitioners to consider the clarity and consistency of their language in the context of athlete development. This article reported evidence of incongruency in the way terms and expressions are defined, understood, and operationalized. The authors highlighted potentially blurry terms and drew attention to potential avenues for future research.

## 3. Final considerations

This research topic aims to facilitate the debate and discussion amongst the sport science community. The ten articles included in this Research Topic “Debates in Elite Sports and Performance Enhancement, 2022” respectfully challenged perspectives and paradigms to enhance sports performance. We believe that the findings and considerations reported in these articles can contribute to the improvement of practitioners' practice and will foster new research questions.

